# Cerebellar involvement in sporadic amyotrophic lateral sclerosis: a magnetic resonance spectroscopy study

**DOI:** 10.1093/braincomms/fcag382

**Published:** 2026-10-12

**Authors:** Gianfranco Vornetti, Francesca Punzetti, Veria Vacchiano, Magali Jane Rochat, Elena Cantoni, Giovanni Rizzo, Diego D’Angeli, Luca Morandi, David Neil Manners, Rocco Liguori, Raffaele Lodi, Caterina Tonon

**Affiliations:** Programma Neuroimmagini Funzionali e Molecolari, IRCCS Istituto delle Scienze Neurologiche di Bologna, Bologna 40139, Italy; Dipartimento di Scienze Biomediche e Neuromotorie, Università di Bologna, Bologna 40138, Italy; Programma Neuroimmagini Funzionali e Molecolari, IRCCS Istituto delle Scienze Neurologiche di Bologna, Bologna 40139, Italy; UOC Clinica Neurologica, IRCCS Istituto delle Scienze Neurologiche di Bologna, Bologna 40139, Italy; Programma Neuroimmagini Funzionali e Molecolari, IRCCS Istituto delle Scienze Neurologiche di Bologna, Bologna 40139, Italy; Programma Neuroimmagini Funzionali e Molecolari, IRCCS Istituto delle Scienze Neurologiche di Bologna, Bologna 40139, Italy; UOC Clinica Neurologica, IRCCS Istituto delle Scienze Neurologiche di Bologna, Bologna 40139, Italy; Dipartimento di Scienze Biomediche e Neuromotorie, Università di Bologna, Bologna 40138, Italy; Programma Neuroimmagini Funzionali e Molecolari, IRCCS Istituto delle Scienze Neurologiche di Bologna, Bologna 40139, Italy; Dipartimento di Scienze Biomediche e Neuromotorie, Università di Bologna, Bologna 40138, Italy; Programma Neuroimmagini Funzionali e Molecolari, IRCCS Istituto delle Scienze Neurologiche di Bologna, Bologna 40139, Italy; Dipartimento di Scienze per la Qualità della Vita, Università di Bologna, Rimini 47921, Italy; Dipartimento di Scienze Biomediche e Neuromotorie, Università di Bologna, Bologna 40138, Italy; UOC Clinica Neurologica, IRCCS Istituto delle Scienze Neurologiche di Bologna, Bologna 40139, Italy; Programma Neuroimmagini Funzionali e Molecolari, IRCCS Istituto delle Scienze Neurologiche di Bologna, Bologna 40139, Italy; Dipartimento di Scienze Biomediche e Neuromotorie, Università di Bologna, Bologna 40138, Italy; Programma Neuroimmagini Funzionali e Molecolari, IRCCS Istituto delle Scienze Neurologiche di Bologna, Bologna 40139, Italy; Dipartimento di Scienze Biomediche e Neuromotorie, Università di Bologna, Bologna 40138, Italy

**Keywords:** amyotrophic lateral sclerosis, cerebellum, magnetic resonance spectroscopy, spastic paraplegia type 4, glutathione

## Abstract

Amyotrophic lateral sclerosis is increasingly recognized as a multisystem neurodegenerative disorder, and recent evidence suggests that the cerebellum may contribute to disease pathology, although cerebellar metabolic alterations have not previously been demonstrated in vivo.

This study investigated cerebellar metabolic and structural changes using proton magnetic resonance spectroscopy (^1^H-MRS) and volumetric imaging. Thirty patients with amyotrophic lateral sclerosis (21 M, mean age 59.7 ± 7.6 years), 22 healthy controls (HC, 10 M, mean age 59.8 ± 9.6 years) and 16 individuals with hereditary spastic paraplegia type 4 (8 M, mean age 50.8 ± 12.5 years), a potential clinical mimic, underwent 3T MRI and ^1^H-MRS with a single-voxel positioned in the left cerebellar hemisphere.

Patients with amyotrophic lateral sclerosis showed significant reductions in myo-inositol/creatinine *(P* = 0.01) and glutathione/creatinine (*P* = 0.005), compared with HC, together with selective volume loss in cerebellar lobules I–IV, V, VI, and Crus I. Among metabolites, glutathione/creatinine had the highest discriminative accuracy for amyotrophic lateral sclerosis versus both HC and spastic paraplegia type 4, with areas under the curve of 0.80 and 0.71, respectively.

These findings reveal distinct cerebellar metabolic alterations in amyotrophic lateral sclerosis, providing further evidence for cerebellar involvement in disease pathology and highlighting cerebellar ^1^H-MRS as a potential adjunct to diagnostic evaluation.

## Teaser

Vornetti *et al*. report that patients with amyotrophic lateral sclerosis show distinct metabolic alterations and selective volume loss in the cerebellum on MRI. These findings provide *in vivo* evidence of cerebellar involvement in disease pathology and support further investigation of cerebellar imaging markers in amyotrophic lateral sclerosis.

## Graphical Abstract

**Graphical Abstract:**
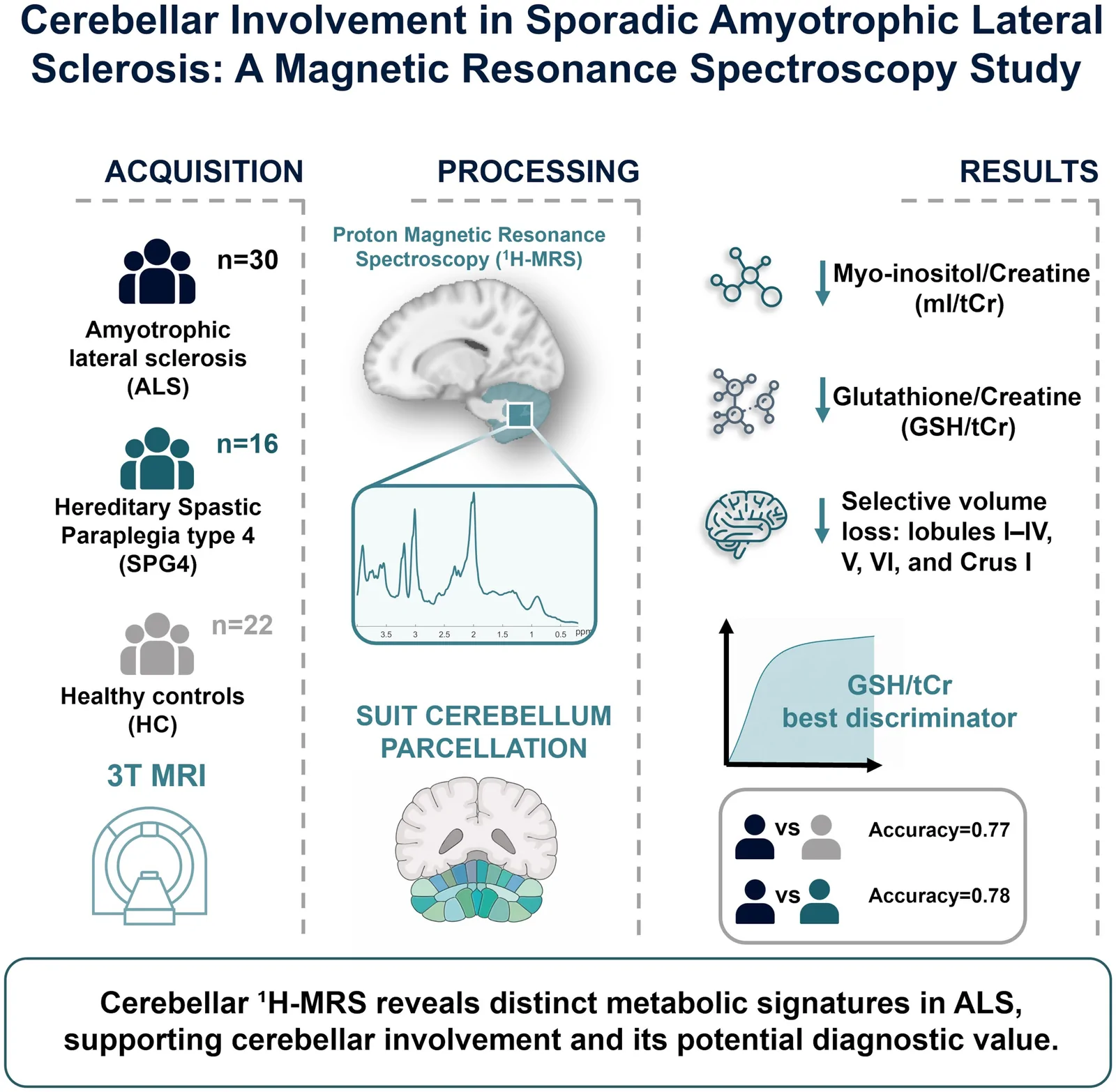

## Introduction

Amyotrophic lateral sclerosis is a progressive neurodegenerative disorder characterized by degeneration of upper and lower motor neurons, leading to muscle weakness, disability and ultimately respiratory failure.^1^ Although classically defined by corticospinal and spinal motor involvement, amyotrophic lateral sclerosis is now increasingly recognized as a multisystem disease with extra-motor pathology demonstrated across neuroimaging, neuropathological and genetic studies.^2^

In this context, the cerebellum has emerged as a potential contributor to amyotrophic lateral sclerosis pathophysiology.^3^ Several structural and functional MRI studies have reported cerebellar atrophy, altered connectivity and changes in cerebellar activation patterns, suggesting that cerebellar circuits may participate in the compensatory or pathological processes associated with amyotrophic lateral sclerosis.^4,5^ Neuropathological studies, particularly in patients carrying intermediate *ATXN2* expansions or *C9orf72* repeat expansions, have reported selective Purkinje cell vulnerability and the presence of abundant p62-positive cerebellar inclusions.^6,7^ A recent transcriptomic study demonstrated widespread RNA dysregulation in the cerebellum of sporadic amyotrophic lateral sclerosis patients, providing molecular evidence of cerebellar involvement even in cases lacking overt cerebellar neuropathology.^8^ Additionally, genetic-association data indicate that amyotrophic lateral sclerosis risk genes are significantly enriched for cerebellar expression, even after excluding genes that are known to cause primary cerebellar diseases, pointing to possible intrinsic vulnerability of the cerebellum in amyotrophic lateral sclerosis.^9^ Despite these observations, the neurometabolic profile of the cerebellum in amyotrophic lateral sclerosis remains largely unexplored.

Proton magnetic resonance spectroscopy (^1^H-MRS) enables *in vivo* quantification of metabolites implicated in neurodegenerative processes and has been widely applied in amyotrophic lateral sclerosis research, predominantly targeting the motor cortex and corticospinal tracts.^10^ However, cerebellar involvement has barely been investigated: to date, only a single study has reported cerebellar ^1^H-MRS findings in amyotrophic lateral sclerosis, examining just six patients and eight controls, and—likely due to limited statistical power—detecting no significant metabolic differences.^11^ Furthermore, no previous studies have provided a combined assessment of cerebellar metabolism and volumetric alterations in amyotrophic lateral sclerosis.

Therefore, the present study aimed to evaluate cerebellar metabolism in patients with amyotrophic lateral sclerosis using single-voxel ^1^H-MRS and to compare these measures with those obtained from healthy controls (HC). Additionally, we evaluated the diagnostic performance of cerebellar metabolite concentrations to explore their potential utility in distinguishing amyotrophic lateral sclerosis not only from HC but also from patients with hereditary spastic paraplegia type 4, the most common autosomal dominant hereditary spastic paraplegia, that can clinically overlap with the early, upper motor neuron–predominant amyotrophic lateral sclerosis phenotype.

## Materials and methods

### Participants

We enrolled consecutive patients diagnosed with probable or definite amyotrophic lateral sclerosis according to the revised El Escorial criteria^12^ (*N* = 30, 21 M and 9 F) who underwent a standardized brain MRI protocol at the IRCCS Institute of Neurological Sciences of Bologna, Italy, between January 2022 and September 2024. Individuals carrying known amyotrophic lateral sclerosis-associated pathogenic mutations were excluded to reduce biological heterogeneity and potential genetic confounding, as emerging evidence suggests that metabolic profiles may differ according to amyotrophic lateral sclerosis genotype.^13^ Two control groups were included: individuals with genetically confirmed spastic paraplegia type 4 (*N* = 16, 8 M and 8 F) who completed brain MRI during the same period, and age-matched HC (*N* = 22, 10 M and 12 F). Collected clinical variables for patients with amyotrophic lateral sclerosis included age at onset, disease duration (time from disease onset to MRI examination), site of symptom onset (bulbar or limb), Amyotrophic Lateral Sclerosis Functional Rating Scale—Revised (ALS-FRS-R) score, Edinburgh Cognitive and Behavioural ALS Screen (ECAS) score and the King’s stage. For spastic paraplegia type 4 patients, we recorded age at onset, disease duration and Spastic Paraplegia Rating Scale (SPRS) scores. All participants provided written informed consent, and the study was approved by the local Ethics Committee (reference number CE 500-2025-OSS-AUSLBO).

### MRI protocol

All MRI examinations were performed on a 3T Siemens MAGNETOM Skyra scanner equipped with a 64-channel head/neck coil. The protocol included a volumetric T1-weighted sequence (3D MPRAGE, magnetization-prepared rapid gradient-echo, sagittal acquisition, isotropic voxel 1 × 1 × 1 mm^3^, no slice gap, TE = 2.98 ms, TR = 2.300 ms, inversion time = 900 ms, flip angle = 9°, acquisition matrix = 256 × 256, pixel bandwidth = 240 Hz, Generalized Autocalibrating Partially Parallel Acquisition (GRAPPA) acceleration factor = 2 and acquisition time ∼ 5 min) and a single-voxel proton MR spectroscopy point-resolved spectroscopy (PRESS) sequence within the left cerebellar hemisphere (Fig. 1, TR = 2000ms, TE = 30 ms, NS = 64, volume = 6 ml, acquisition time = 2 min 18 s).

**Figure 1:**
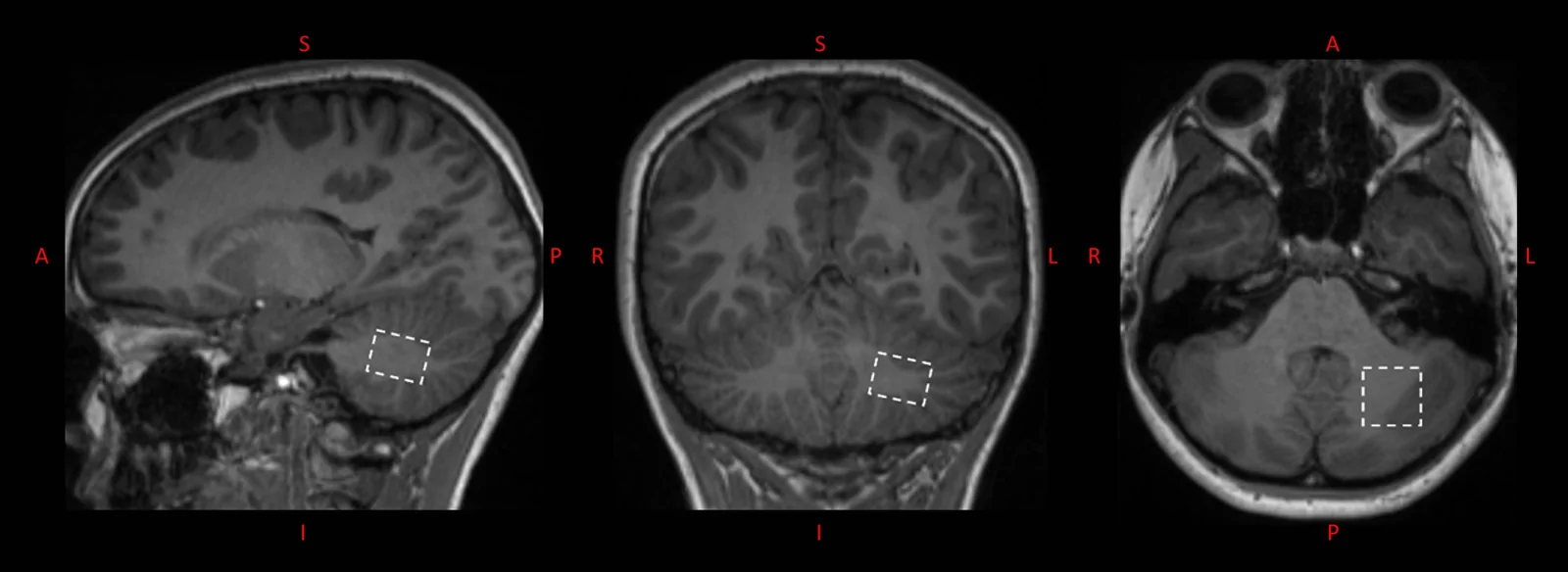
**Representative ^1^H-MRS volume of interest localization within the left cerebellar hemisphere of a 51-year-old HC.** PRESS sequence TR = 2000 ms, TE = 30 ms, NS = 64, volume = 6 ml, acquisition time = 2 min 18 s. HC, healthy controls; ^1^H-MRS, proton magnetic resonance spectroscopy; PRESS, point-resolved spectroscopy; TR, repetition time; TE, echo time; NS, number of scans; A, anterior; P, posterior; I, inferior; S, superior; L, left; R, right.

### Quality assessment and metabolites quantification

Spectral quality was assessed through initial visual inspection followed by quantitative criteria. Only spectra with a signal-to-noise ratio ≥12 and a full width at half maximum ≤0.08 ppm were included, in accordance with established recommendations.^14^ The signal-to-noise ratio was calculated as the ratio between the maximum signal intensity of the baseline-corrected spectrum within the selected ppm range and twice the root mean square of the residuals. Metabolite concentrations were quantified using LCModel v. 6.3.^15^ Spectra were fitted between 4.0 and 0.2 ppm using a pre-generated LCModel-compatible PRESS basis set matched to the acquisition parameters.

Eddy-current correction was performed using the unsuppressed water reference signal. The fitted model provided estimates for *N*-acetyl-aspartate (NAA), choline-containing compounds (Cho), myo-inositol (mI), glutamate (Glu) and glutathione (GSH), expressed as ratios relative to creatinine (tCr).

Spectral referencing and zero- and first-order phase parameters were optimized within LCModel using default settings; no additional frequency or phase correction was applied before fitting.

Macromolecular and lipid resonances were modelled by separate MM/Lip components reported in the fit output, although no experimentally measured subject-specific macromolecular spectrum was included in the basis set.

Cramér–Rao lower bounds (CRLBs), expressed as percentage standard deviations, were used as metabolite-specific indicators of quantification precision; with a strict inclusion threshold set at <15% for all evaluated metabolites, lower CRLB values indicate more reliable metabolite estimates.

No explicit voxel-wise correction for chemical-shift displacement was applied; however, voxel placement was carefully optimized within the left cerebellar hemisphere, and to account for differences in grey matter (GM), white matter (WM) and cerebrospinal fluid (CSF) content within the spectroscopy voxel, tissue fractions were calculated from the T1-weighted images using FMRIB Software Library (FSL)-MRS, as previously reported.^16^

Metabolite ratios were not corrected for T1 or T2 relaxation effects and should therefore be interpreted as apparent ratios under the acquisition and fitting conditions used in this study.

To provide a visual assessment of spectral quality and fitting performance, mean spectra for each cohort, including the measured spectrum, LCModel fit and LCModel fit ± SD, are reported in Fig. 2.

**Figure 2:**
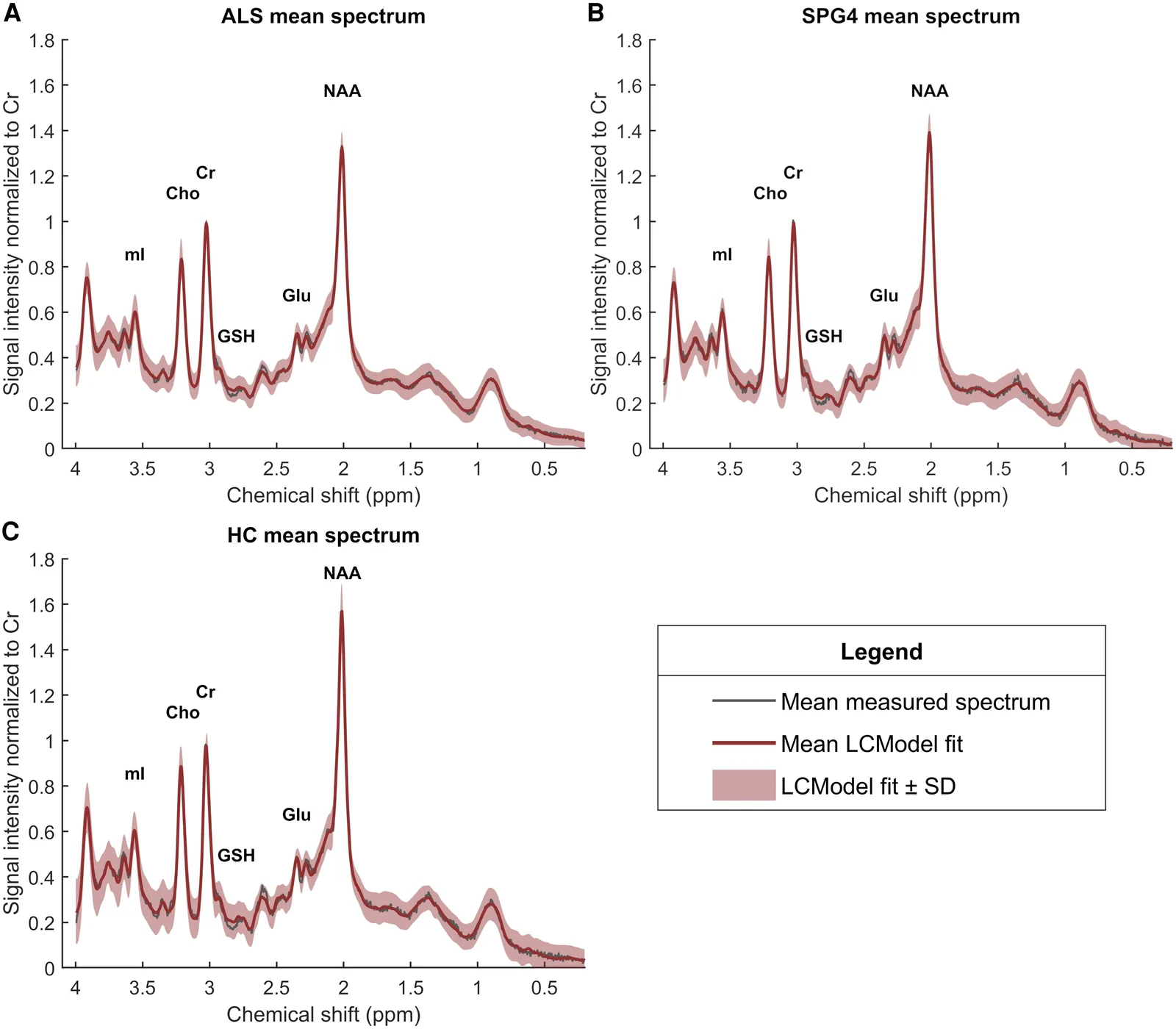
**Mean spectra from the left cerebellar hemisphere are shown for amyotrophic lateral sclerosis (A, sample size = 21), spastic paraplegia type 4 (B, sample size = 22) and healthy control (C, sample size = 16) groups.** The grey line represents the mean measured spectrum, the red line represents the mean LCModel fit and the shaded red area represents the LCModel fit ± SD. Main metabolite resonances are labelled. ALS, amyotrophic lateral sclerosis; SPG4, hereditary spastic paraplegia type 4; HC, healthy controls; ppm, parts per million; NAA, *N*-acetyl-aspartate; Cr, creatinine; Cho, choline-containing compounds; mI, myo-inositol; Glu, glutamate; GSH, glutathione.

### Cerebellar volumetry

Cerebellar volumetry was performed using the Spatially Unbiased Infratentorial Template (SUIT) toolbox,^17^ which provides high-resolution normalization and segmentation optimized for the cerebellum and brainstem. T1-weighted images were first isolated to extract the infratentorial structures, removing cortical and subcortical supratentorial tissue using the SUIT isolation procedure. The resulting cerebellar masks were then segmented into individual lobules using the probabilistic atlas implemented in SUIT, and bilateral volumes were calculated for each. Total intracranial volume (TIV) was estimated for each subject using FreeSurfer v. 6 to account for inter-individual differences in head size.

### Statistical analysis

Because the amyotrophic lateral sclerosis, HC and spastic paraplegia type 4 groups differed in age and sex distribution, and since metabolite levels may be affected by voxel tissue composition, all metabolite ratios were corrected for age, sex and GM/WM/CSF fractions. Correction parameters were derived through ordinary least squares (OLS) regression using data from the HC group, implemented with the statsmodels v. 0.14.0 library in Python, and subsequently applied to the amyotrophic lateral sclerosis and spastic paraplegia type 4 datasets. The distribution of OLS residuals was assessed using the Shapiro–Wilk test. When residuals followed a normal distribution, group comparisons were performed using one-way ANOVA followed by Tukey’s HSD test. When normality assumptions were not met, the Kruskal–Wallis test was used, followed by Dunn’s *post hoc* test. All *P*-values were corrected for multiple comparisons using the false discovery rate (FDR), and values <0.05 (two-tailed) were considered statistically significant.

To investigate the potential diagnostic value of cerebellar metabolites in distinguishing amyotrophic lateral sclerosis from both HC and spastic paraplegia type 4, receiver operating characteristic (ROC) analyses were performed. For each metabolite showing significant between-group differences, the area under the curve (AUC) was calculated, and the optimal classification threshold was determined using the Youden index, from which corresponding sensitivity and specificity values were derived.

## Results

### Study population

All spectra of 30 patients with sporadic amyotrophic lateral sclerosis (21 M; mean age 59.7 ± 7.6 years), 22 HC (10 M; mean age 59.8 ± 9.6 years) and 16 patients with spastic paraplegia type 4 (8 M; mean age 50.8 ± 12.5 years) were included. spastic paraplegia type 4 patients were younger compared to both amyotrophic lateral sclerosis patients and HC (*P* = 0.018 and *P* = 0.055, respectively), while no age difference was found between the amyotrophic lateral sclerosis and HC groups (*P* = 0.56). Among individuals with amyotrophic lateral sclerosis, 6 (20%) presented with bulbar onset. The mean disease duration at the time of MRI was 1.2 ± 0.6 years. According to the King’s staging, 13% were in stage I, 40.0% in stage II, 40.0% in stage III and 7% in stage IV. The mean ALS-FRS-R score was 41 ± 3. In the spastic paraplegia type 4 group, the mean disease duration was 24 ± 22 years, and the mean SPRS score was 14 ± 10 (demographic and clinical characteristics of the study population are reported in Table 1).

**Table 1:** Demographic and clinical characteristics of the study population

Group	Amyotrophic lateral sclerosis	Spastic paraplegia type 4	HC
*N*	30 (21 M)	16 (8 M)	22 (10 M)
Age (years)	59.7 ± 7.6	50.8 ± 12.5	59.8 ± 9.6
Age at onset (years)	59.6 ± 6.4	27.4 ± 18.16	
Disease duration (years)	1.2 ± 0.6	24 ± 22	
SPRS		14 ± 10	
ALS-FRS-R	41 ± 3		
King’s stage (I:II:III:IV)	4:12:12:2		
Bulbar onset	8		
ECAS total score	107 ± 14 (6 NA)		
Survival (years)	1.8 ± 0.96 (15 NA)	NA	

Disease duration was calculated from symptom onset to MRS acquisition. King’s clinical stage is reported as the number of amyotrophic lateral sclerosis patients in each stage. Survival is reported for patients with available follow-up data; the number of missing values is indicated in parentheses.

ALS, amyotrophic lateral sclerosis; SPG4, hereditary spastic paraplegia type 4; HC, healthy controls; M, males; SPRS, Spastic Paraplegia Rating Scale; ALS-FRS-R, Amyotrophic Lateral Sclerosis Functional Rating Scale–Revised; ECAS, Edinburgh Cognitive and Behavioural ALS Screen; NA, not available; MRS, magnetic resonance spectroscopy.

### Metabolite concentrations

Group comparisons of cerebellar metabolite ratios are summarized in Fig. 3. The most pronounced difference between amyotrophic lateral sclerosis and HC was observed for GSH/tCr, which decreased from 0.34 ± 0.07 in HC to 0.27 ± 0.05 in amyotrophic lateral sclerosis (*P* = 0.005). Compared with HC, patients with amyotrophic lateral sclerosis also showed significantly reduced levels of mI/tCr, which decreased from 0.78 ± 0.10 in HC to 0.67 ± 0.11 in amyotrophic lateral sclerosis (*P* = 0.01). Cho/tCr and NAA/tCr also showed a slight reduction in amyotrophic lateral sclerosis patients compared with HC (0.31 ± 0.04 versus 0.29 ± 0.05 and 1.36 ± 0.27 versus 1.46 ± 0.41), which did not reach statistical significance (*P* = 0.16 and *P* = 0.22, respectively).

**Figure 3:**
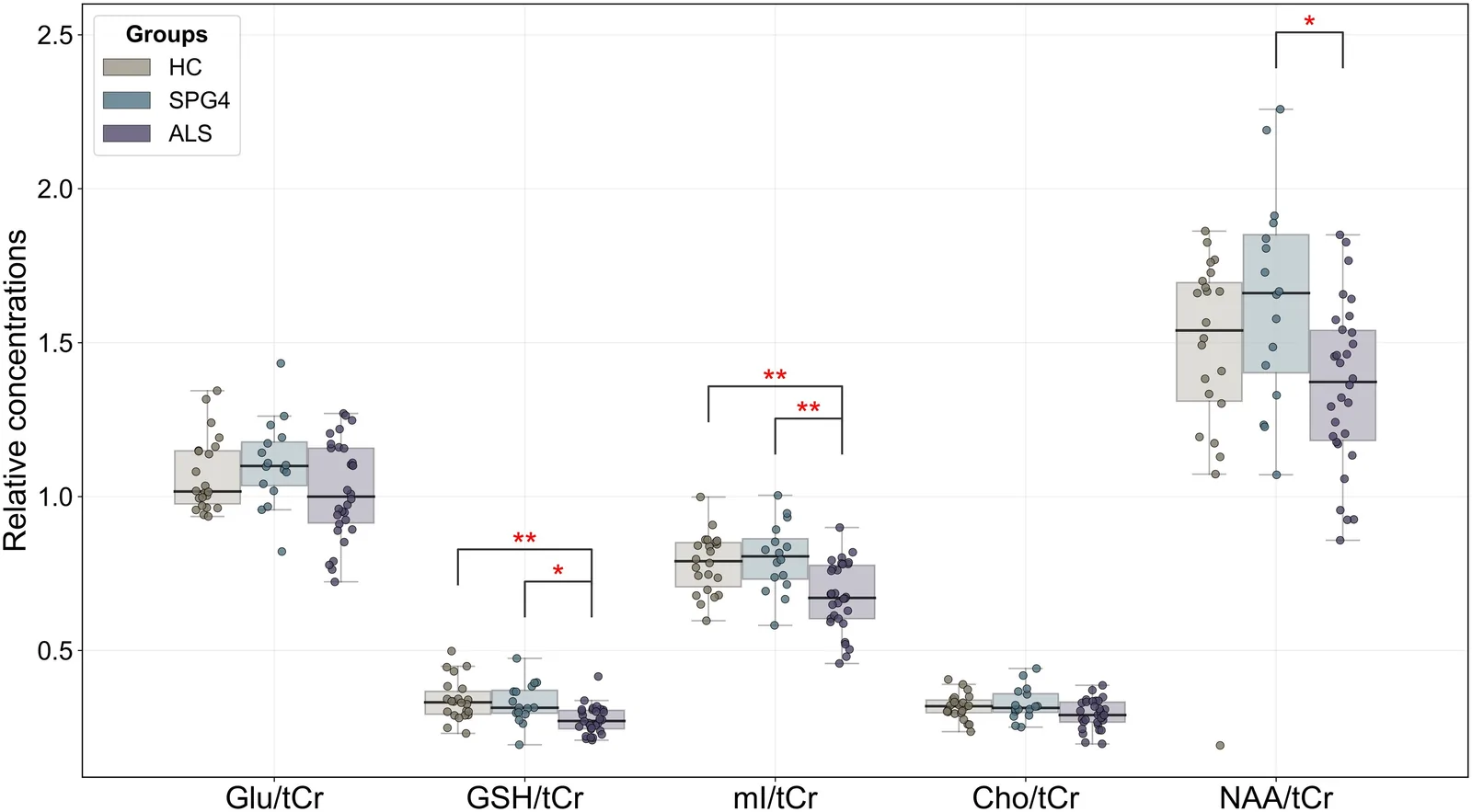
**Boxplot representing the distribution of metabolite concentrations relative to creatinine (Cr).** Values were adjusted for sex, age and tissue fractions in the spectroscopy voxel. Significantly different distributions within groups are highlighted in red, considering corrected *P*-values (ANOVA or Kruskal–Wallis tests, *: 0.01 < *P*-value < 0.05, **: 0.001 < *P*-value ≤ 0.01). Each dot represents one participant. Sample size: amyotrophic lateral sclerosis = 30, spastic paraplegia type 4 = 16, HC = 22. ALS, amyotrophic lateral sclerosis; SPG4, hereditary spastic paraplegia type 4; HC, healthy controls; Glu, glutamate; GSH, glutathione; mI, myo-inositol; Cho, choline; NAA, *N*-acetyl-aspartate; Cr, creatinine.

A similar pattern was observed when comparing amyotrophic lateral sclerosis with spastic paraplegia type 4 patients. amyotrophic lateral sclerosis patients exhibited lower mI/tCr (0.67 ± 0.11 versus 0.80 ± 0.11, *P* = 0.014) and GSH/tCr (0.29 ± 0.05 versus 0.33 ± 0.05, *P* = 0.024), as well as a reduction in NAA/tCr values (1.36 ± 0.27 versus 1.66 ± 0.34, *P* = 0.017). Cho/tCr was also reduced in amyotrophic lateral sclerosis patients compared with the spastic paraplegia type 4 group (0.29 ± 0.05 versus 0.33 ± 0.05), although without reaching statistical significance (*P* = 0.16).

No significant differences in cerebellar metabolite ratios were found between spastic paraplegia type 4 patients and HC (the full comparison of cerebellar metabolite levels across the three groups is reported in Supplementary Table 1).

Exploratory analyses using water-referenced metabolite concentrations are reported in Supplementary Table 2. For the comparisons that were statistically significant using tCr as the reference, the direction of the effect was preserved with water referencing. However, several corresponding comparisons did not remain significant after FDR correction, and some did not reach nominal significance. Among other comparisons that were not significant using tCr, the direction of the effect occasionally differed between the two reference methods.

No significant associations were observed between any cerebellar metabolite ratios and clinical severity measures, disease duration or site of symptom onset (bulbar versus limb) in amyotrophic lateral sclerosis patients, nor between metabolite ratios and either disease duration or SPRS scores in the spastic paraplegia type 4 group.

### Classification analysis

Among metabolites showing significant group differences, GSH/tCr demonstrated the highest discriminative accuracy for both amyotrophic lateral sclerosis versus HC and amyotrophic lateral sclerosis versus spastic paraplegia type 4 (Table 2). For distinguishing amyotrophic lateral sclerosis from HC, GSH/tCr yielded an AUC of 0.80, with a sensitivity of 0.87 and a specificity of 0.64 at the optimal classification threshold of 0.31. For amyotrophic lateral sclerosis versus spastic paraplegia type 4, GSH/tCr achieved an AUC of 0.71, with a sensitivity of 0.87 and a specificity of 0.62 at the same optimal threshold of 0.31.

**Table 2:** Classification performances

Metabolite	Groups	AUC	Cut off	Specificity	Sensitivity	Accuracy
GSH/Cr	Amyotrophic lateral sclerosis versus HC	0.802	0.310	0.636	0.867	0.769
	Amyotrophic lateral sclerosis versus spastic paraplegia type 4	0.707	0.310	0.625	0.867	0.783
mI/Cr	Amyotrophic lateral sclerosis versus HC	0.745	0.687	0.773	0.633	0.692
	Amyotrophic lateral sclerosis versus spastic paraplegia type 4	0.733	0.686	0.875	0.633	0.717
Cho/Cr	Amyotrophic lateral sclerosis versus HC	0.641	0.291	0.773	0.533	0.635
	Amyotrophic lateral sclerosis versus spastic paraplegia type 4	0.633	0.304	0.750	0.600	0.652
NAA/Cr	Amyotrophic lateral sclerosis versus HC	0.639	1.464	0.591	0.667	0.635
	Amyotrophic lateral sclerosis versus spastic paraplegia type 4	0.713	1.575	0.688	0.800	0.761

Summary of ROC curve for the metabolites showing significant differences between groups.

GSH, glutathione; mI, myo-inositol; Cho, choline; NAA, *N*-acetyl-aspartate; Cr, creatinine.

### Cerebellar volumetry

Cerebellar volumetric analysis revealed selective lobular atrophy in the amyotrophic lateral sclerosis group compared with HC. After adjusting for TIV and correcting for multiple comparisons, patients with amyotrophic lateral sclerosis showed significantly reduced volumes in both anterior and posterior cerebellar regions. Specifically, amyotrophic lateral sclerosis patients exhibited lower volumes in lobules I–IV (*P* = 0.047), lobule V (*P* = 0.040), lobule VI (*P* = 0.040) and Crus I (*P* = 0.033) compared with HC.

In contrast, no significant volumetric differences were observed between the amyotrophic lateral sclerosis and spastic paraplegia type 4 groups, nor between spastic paraplegia type 4 patients and HC.

## Discussion

In this study, we provide new evidence for cerebellar involvement in amyotrophic lateral sclerosis by demonstrating significant neurometabolic alterations on cerebellar ^1^H-MRS in the largest cohort investigated to date using this technique, together with selective volumetric loss across multiple cerebellar lobules. These metabolic and structural changes suggest that the cerebellum is not merely engaged in compensatory mechanisms but is itself affected by disease-related pathology.

The metabolic profile we observed differs from the canonical pattern described in neurodegenerative disorders,^18^ including in the motor cortex and corticospinal tracts of amyotrophic lateral sclerosis patients, which is typically characterized by reduced NAA alongside elevations in mI, a pattern interpreted as neuronal loss combined with reactive gliosis.^10^ In contrast, our cerebellar findings reveal a reduction in mI/tCr, with a smaller, statistically non-significant reduction in NAA/tCr.

Myo-inositol is a precursor of phosphatidylinositol and downstream inositol-phosphate signalling molecules and also serves as a major osmolyte in the brain. It is highly concentrated in astrocytes and is therefore widely regarded as an *in vivo* marker of glial density and function.^19^ Elevated mI is commonly reported across neurodegenerative conditions, including in amyotrophic lateral sclerosis motor regions, where it is considered one of the spectroscopic hallmarks of reactive gliosis.^10^ In contrast, reduced mI may indicate impaired osmolyte regulation and astrocyte loss or dysfunction.

Nevertheless, the observed mI/tCr reduction should be interpreted with caution because no macromolecular component specifically accounted for signal overlapping the mI spectral region during LCModel fitting. Unmodelled macromolecular contributions may therefore have influenced the estimated mI levels.

Structural imaging studies have shown volume reductions in both anterior and posterior cerebellar lobules in amyotrophic lateral sclerosis patients.^4^ This pattern of atrophy was confirmed in our cohort, where lobules I–IV, V, VI and Crus I showed significant volume loss relative to controls. While cerebellar atrophy is traditionally attributed to neuronal degeneration, the neurometabolic profile we observed raises the possibility that glial rather than neuronal loss may be a contributing factor. Astrocytes constitute a substantial proportion of cerebellar cellular volume, particularly within the dense astroglial networks formed by Bergmann glia in the Purkinje layer and astrocytes in the granular layer.^20^ Thus, astrocytic degeneration might lead not only to reduced concentrations of astrocyte-enriched metabolites but also to measurable tissue volume loss.

Although there is a scarcity of post-mortem studies evaluating cerebellar pathology in amyotrophic lateral sclerosis patients,^21,22^ the presence of TDP-43 inclusions has been described both in cerebellar neurons and astrocytes.^23^ Additionally, animal models of amyotrophic lateral sclerosis, such as SOD1(G93A) transgenic mice, have demonstrated PARP activation in astrocytes in cerebellar nuclei,^24^ which in turn may lead to ATP depletion and eventual cell death.^25^

The observed reduction in cerebellar GSH/tCr in our cohort of amyotrophic lateral sclerosis patients aligns with previous research, which has found oxidative stress to be a major pathological mechanism in amyotrophic lateral sclerosis, including within the cerebellum.^26^ As the brain’s major endogenous antioxidant, GSH is particularly enriched in astrocytes, which play a central role in buffering reactive oxygen species and supplying neurons with GSH precursors.^27,28^ The reduction in cerebellar GSH observed in our amyotrophic lateral sclerosis cohort may therefore further support the hypothesis of astrocyte loss or dysfunction within the cerebellum.

Regarding the potential diagnostic utility of cerebellar ^1^H-MRS, GSH/tCr emerged as the most informative metabolite ratio, achieving fair discriminative performance for both amyotrophic lateral sclerosis versus HC and amyotrophic lateral sclerosis versus spastic paraplegia type 4. Our results show a moderate improvement over conventional MRI markers, such as corticospinal tract T2/FLAIR hyperintensity or motor cortex T2*/SWI hypointensity^29^ but do not reach the highest diagnostic accuracy reported for advanced diffusion or susceptibility-based metrics in distinguishing amyotrophic lateral sclerosis from either HC or potential mimics.^30,31^ Nonetheless, unlike these advanced imaging techniques, cerebellar ^1^H-MRS requires a much shorter acquisition time (∼2 min in our protocol) and can therefore be readily integrated into routine MRI exams, potentially offering a practical adjunct for improving diagnostic evaluation in clinical practice.

In our study, no significant associations were observed between cerebellar metabolite levels and clinical measures or disease duration. This lack of correlation has been reported in other cerebellar imaging studies using different modalities and may indicate that cerebellar pathology progresses somewhat independently of motor neuron degeneration.^3,21^ Additionally, current clinical scales also primarily capture limb and bulbar motor function and may not sensitively reflect cerebellar involvement.

This study has several limitations. First, although this represents the largest cerebellar ^1^H-MRS cohort in amyotrophic lateral sclerosis to date, the overall sample size remains modest, preventing subgroup comparisons due to limited statistical power. Additionally, although we observed no significant relationships between metabolite concentrations and disease duration or stage, the cross-sectional design precludes inferences regarding temporal progression of cerebellar metabolic changes.

A further potential limitation is the use of tCr as an internal reference, as its concentration may be altered, influencing metabolite ratio estimates. Although no significant group difference in tCr concentration was detected, exploratory water-referenced analyses showed reduced statistical robustness. For the comparisons that were significant using tCr, the direction of effect was preserved with water referencing, but several comparisons did not remain significant after FDR correction and some did not reach nominal significance. Directional differences between reference methods were also observed for some other non-significant comparisons. These findings indicate that the choice of reference affected the magnitude and statistical robustness of the group differences and that the tCr-referenced findings should therefore be interpreted cautiously. In addition, metabolite ratios were not corrected for T1 or T2 relaxation effects; differences in relaxation properties between metabolites or participant groups may consequently have introduced bias into the apparent ratios.

Finally, spectral acquisition was restricted to a single voxel in the left cerebellar hemisphere, which may not fully capture spatial heterogeneity of cerebellar involvement.

## Conclusion

Overall, this is the first study to demonstrate significant *in vivo* cerebellar neurometabolic alterations in amyotrophic lateral sclerosis, providing further evidence that the cerebellum is actively involved in amyotrophic lateral sclerosis pathophysiology. The observed reductions in astrocyte-enriched metabolites suggest that cerebellar astrocytic dysfunction or loss may play a role in disease mechanisms, which will need to be confirmed in dedicated neuropathological studies. From a diagnostic perspective, cerebellar ^1^H-MRS emerges as a practical adjunct to routine MRI, requiring only minimal additional scan time and providing complementary information that could contribute to multimodal diagnostic assessment. Larger prospective studies are necessary to confirm its diagnostic utility and its applicability across diverse clinical contexts.

## Supplementary Material

fcag382_Supplementary_Data

## Data Availability

The per-subject data underlying this article and the code used for statistical analysis are deposited in Zenodo repository (https://zenodo.org) and are accessible with the following link: https://doi.org/10.5281/zenodo.20798577.
