# Supplementary material for: Cerebellar involvement in sporadic amyotrophic lateral sclerosis: a magnetic resonance spectroscopy study
Source: Brain Commun. 2026 Oct 12;8(5):fcag382. doi: 10.1093/braincomms/fcag382 (PMC13690432; doi:10.1093/braincomms/fcag382)
Supplement: Supplementary file 1 [file fcag382_supplementary_data.docx]

**Supplementary Material**

**Statistical analysis results – Concentration relative to tCr**

| Group1 | direction | Group2 | Variables | P uncorr | P groups corrected | P tot corrected | Significance | Z | Effect size |
| --- | --- | --- | --- | --- | --- | --- | --- | --- | --- |
| ALS | **<** | **HC** | Cho/tCr | 0.084186 | 0.126278 | 0.189418 |  | -1.7269 | -0.28788 |
| ALS | **<** | **HC** | NAA/tCr | 0.112978 | 0.161508 | 0.22353 |  | -1.58495 | -0.27576 |
| ALS | **<** | **HC** | Glu/tCr | 0.316415 | 0.316415 | 0.406819 |  | -1.00185 | -0.2 |
| ALS | **<** | **HC** | mI/tCr | 0.001255 | 0.003566 | 0.011293 | * | -3.22613 | -0.53939 |
| ALS | **<** | **HC** | GSH/tCr | 0.000287 | 0.000862 | 0.005175 | ** | -3.62633 | -0.6 |
| ALS | **<** | **SPG4** | Glu/tCr | 0.03169 | 0.095069 | 0.095069 |  | -2.1483 | -0.35111 |
| ALS | **<** | **SPG4** | Cho/tCr | 0.061331 | 0.126278 | 0.157708 |  | -1.8711 | -0.34667 |
| ALS | **<** | **SPG4** | mI/tCr | 0.002377 | 0.003566 | 0.014264 | * | -3.03854 | -0.56 |
| ALS | **<** | **SPG4** | NAA/tCr | 0.003861 | 0.011584 | 0.017376 | * | -2.88928 | -0.52 |
| ALS | **<** | **SPG4** | GSH/tCr | 0.006768 | 0.010152 | 0.024366 | * | -2.70803 | -0.50667 |
| HC | **<** | **SPG4** | NAA/tCr | 0.161508 | 0.161508 | 0.264286 |  | -1.40002 | -0.30303 |
| HC | **<** | **SPG4** | Glu/tCr | 0.234428 | 0.316415 | 0.324592 |  | -1.18903 | -0.30303 |
| HC | **>** | **SPG4** | GSH/tCr | 0.629532 | 0.629532 | 0.755438 |  | 0.482386 | 0.09697 |
| HC | **<** | **SPG4** | Cho/tCr | 0.749382 | 0.749382 | 0.843054 |  | -0.31945 | -0.06667 |
| HC | **<** | **SPG4** | mI/tCr | 0.868771 | 0.868771 | 0.903829 |  | -0.16522 | -0.04242 |

**Supplementary Table 1: Analysis results for metabolites concentration relative to tCr**

**Statistical analysis results – Concentration relative to water reference**

| Group1 | direction | Group2 | Variables | P uncorr | P groups corrected | P tot corrected | Significance | Z | Effect size |
| --- | --- | --- | --- | --- | --- | --- | --- | --- | --- |
| ALS | **<** | **HC** | Glu | 0.084723 | 0.129799 | 0.194698 |  | -1.72392 | -0.38182 |
| ALS | **<** | **HC** | GSH | 0.010733 | 0.032198 | 0.064397 |  | -2.55128 | -0.55909 |
| ALS | **<** | **HC** | Ins | 0.010573 | 0.03172 | 0.064397 |  | -2.55649 | -0.51818 |
| ALS | **<** | **HC** | Cho | 0.131516 | 0.332052 | 0.263033 |  | -1.50815 | -0.32273 |
| ALS | **<** | **HC** | NAA | 0.001492 | 0.004476 | 0.026853 | * | -3.17627 | -0.7 |
| ALS | **<** | **HC** | tCr | 0.41099 | 0.503662 | 0.569062 |  | -0.82215 | -0.16818 |
| ALS | **>** | **SPG4** | Glu | 0.903758 | 0.903758 | 0.903758 |  | 0.120916 | 0.046667 |
| ALS | **<** | **SPG4** | GSH | 0.599157 | 0.599157 | 0.718988 |  | -0.52561 | -0.10667 |
| ALS | **>** | **SPG4** | Ins | 0.878402 | 0.878402 | 0.903758 |  | 0.152996 | -0.08 |
| ALS | **>** | **SPG4** | Cho | 0.868681 | 0.868681 | 0.903758 |  | 0.165334 | -0.04 |
| ALS | **<** | **SPG4** | NAA | 0.270006 | 0.270006 | 0.405009 |  | -1.10305 | -0.23333 |
| ALS | **>** | **SPG4** | tCr | 0.503662 | 0.503662 | 0.647566 |  | 0.668738 | 0.146667 |
| HC | **>** | **SPG4** | Glu | 0.086532 | 0.129799 | 0.194698 |  | 1.713979 | 0.381818 |
| HC | **>** | **SPG4** | GSH | 0.069084 | 0.103626 | 0.194698 |  | 1.817871 | 0.406061 |
| HC | **>** | **SPG4** | Ins | 0.027611 | 0.041416 | 0.124249 |  | 2.202773 | 0.551515 |
| HC | **>** | **SPG4** | Cho | 0.221368 | 0.332052 | 0.362239 |  | 1.222898 | 0.284848 |
| HC | **>** | **SPG4** | NAA | 0.070994 | 0.106491 | 0.194698 |  | 1.805514 | 0.393939 |
| HC | **>** | **SPG4** | tCr | 0.149655 | 0.448966 | 0.26938 |  | 1.44075 | 0.345455 |

**Supplementary Table 2: Analysis results for metabolites concentration relative to water reference**
